# Differential expression of c-Met between primary and metastatic sites in clear-cell renal cell carcinoma and its association with PD-L1 expression

**DOI:** 10.18632/oncotarget.21952

**Published:** 2017-10-23

**Authors:** Aly-Khan A. Lalani, Kathryn P. Gray, Laurence Albiges, Marcella Callea, Jean-Christophe Pignon, Soumitro Pal, Mamta Gupta, Rupal S. Bhatt, David F. McDermott, Michael B. Atkins, G.F. Vande Woude, Lauren C. Harshman, Toni K. Choueiri, Sabina Signoretti

**Affiliations:** ^1^ Lank Center for Genitourinary Oncology, Dana-Farber Cancer Institute, Boston, MA, USA; ^2^ Department of Biostatistics and Computational Biology, Dana-Farber Cancer Institute, Boston, MA, USA; ^3^ Institut Goustave Roussy, Villejuif, France; ^4^ Department of Pathology, Ospedale San Raffaele, Milan, Italy; ^5^ Department of Pathology, Brigham and Women’s Hospital, Boston, MA, USA; ^6^ Division of Nephrology, Boston Children’s Hospital, Boston, MA, USA; ^7^ Department of Pathology, Beth Israel Deaconess Medical Center, Boston, MA, USA; ^8^ Division of Hematology/Oncology, Beth Israel Deaconess Medical Center, Boston, MA, USA; ^9^ Georgetown Lombardi Comprehensive Cancer Center, Washington D.C., USA; ^10^ Van Andel Research Institute, Grand Rapids, MA, USA

**Keywords:** c-Met, PD-L1, primary, metastasis, renal cell carcinoma

## Abstract

In preclinical models, c-Met promotes survival of renal cancer cells through the regulation of programmed death-ligand 1 (PD-L1). However, this relationship in human clear cell renal cell carcinoma (ccRCC) is not well characterized. We evaluated c-Met expression in ccRCC patients using paired primary and metastatic samples and assessed the association with PD-L1 expression and other clinical features. Areas with predominant and highest Fuhrman nuclear grade (FNG) were selected. c-Met expression was evaluated by IHC using an anti-Met monoclonal antibody (MET4 Ab) and calculated by a combined score (CS, 0–300): intensity of c-Met staining (0–3) x % of positive cells (0–100). PD-L1 expression in tumor cells was previously assessed by IHC and PD-L1+ was defined as PD-L1 > 0% positive cells. Our cohort consisted of 45 pairs of primary and metastatic ccRCC samples. Overall, c-Met expression was higher in metastatic sites compared to primary sites (average c-Met CS: 55 vs. 28, *p* = 0.0003). Higher c-Met expression was associated with higher FNG (4 vs. 3) in primary tumors (average c-Met CS: 52 vs. 20, *p* = 0.04). c-Met expression was numerically greater in PD-L1+ vs. PD-L1- tumors. Higher c-Met expression in metastatic sites compared to primary tumors suggests that testing for biomarkers of response to c-Met inhibitors should be conducted in metastases. While higher c-Met expression in PD-L1+ tumors requires further investigation, it supports exploring these targets in combination clinical trials.

## INTRODUCTION

The cell-surface receptor tyrosine kinase c-Met is encoded by the *c-MET* proto-oncogene and is involved in several key functions, including cell growth, cell differentiation, neo-vascularization, and tissue repair [1]. c-Met and its only known ligand, hepatocyte growth factor (HGF), have been implicated in tumor development, invasion, migration and angiogenesis in solid tumor malignancies, including renal cell carcinoma (RCC) [2, 3]. The prognostic relevance of c-Met expression has been explored in several tumor types and shown to be associated with poorer outcomes [4, 5]. In RCC, high c-Met expression was reported to be an independent predictor of survival in 330 nephrectomy cases using quantitative immunofluorescence [6]. In the metastatic RCC (mRCC) setting, c- Met expression also appears to be associated with worse outcomes in a retrospective cohort of patients treated with sunitinib, an antiangiogenic agent against vascular endothelial growth factor receptor (VEGFR) [7].

C-Met and the tyrosine-kinase AXL can be upregulated in RCC and have been shown to play a possible role in the development of resistance to VEGFR inhibitors making these pathways rational targets for therapeutic trials [8–13]. Cabozantinib, an oral, small-molecule inhibitor of VEGFR, MET, and AXL, improved overall survival compared to everolimus in previously-treated patients with advanced RCC [14, 15]. A Phase II study also showed the promise of cabozantinib to improve progression-free survival (PFS) and response rates in patients with untreated intermediate and poor risk mRCC compared to standard of care sunitinib [16]. With the expanding treatment armament and the likely importance of c-Met in controlling mRCC, the quest for an optimal model to assess for predictive biomarkers for c-Met inhibition has emerged.

The HGF/c-Met pathway has also been implicated in attenuating inflammatory responses, which suggests potential for immunomodulation with inhibition of this pathway [17, 18]. Preclinical models have shown that c-Met expression promotes the upregulation of programmed death-ligand 1 (PD-L1) and that this increase protects renal cancer cells from immune-mediated cytotoxicity [19]. However, the relationship between c-Met and PD-L1 in human mRCC has not been well characterized.

In this context, and given the known tumoral heterogeneity in this disease [20], we aimed to compare the expression of c-Met between paired primary and metastatic sites in clear-cell RCC (ccRCC) tissues. We also evaluated the potential association of c-Met expression with clinicopathological factors and PD-L1 expression in tumor cells in both primary and metastatic sites. In a descriptive analysis of a subset of patients treated with VEGF targeted therapy (VEGF-TT), we report on the effect of c-Met status on clinical outcomes and the effect of treatment in between primary and metastatic sampling on c-Met expression.

## RESULTS

### Patient population and tumor tissue selection

We identified 45 patients with both primary and metastatic lesions available for analysis. Patient characteristics at the time of primary surgery are summarized in Table 1. Median age was 58 and 64% of patients were male. Pathologic T-stage at diagnosis was T1/T2 in 17 (38%) patients and T3/T4 in 25 (56%). No FNG I or II were reported in the cohort; 32 patients had FNG III and 13 had FNG IV. Metastatic sites included: lung (*n =* 14), bone (*n =* 4), lymph nodes (*n =* 10), soft tissues (*n =* 5), adrenal gland (*n =* 6), pleura (*n =* 3), brain (*n =* 1), thyroid (*n =* 1), and others (*n =* 10). Although most primary tumors had only one corresponding metastasis, 10 cases (22%) had two or more metastatic lesions that could be retrieved, which resulted in a total of 54 metastases that were analyzed.

**Table 1:** Patient characteristics at primary surgery

	Total *n =* 45
Characteristics	Patients, *n* (%)
Gender	
Male	29 (64)
Female	16 (36)
Median age at primary surgery, y (range)	58 (49–62)
T stage	
T1	3 (7)
T2	14 (31)
T3	21 (47)
T4	4 (9)
Tx	3 (7)
N stage	
N0	12 (27)
N1	13 (29)
Nx	20 (44)
Fuhrman nuclear grade (FNG)	
III	32 (71)
IV	13 (29)
Number of metastatic sites analyzed per case	
1	35 (78)
2	6 (13)
≥ 3	4 (9)
Systemic therapy	
VEGF targeted therapy (VEGF-TT)	20 (44)
Vaccine-based or other therapies	16 (36)
No systemic therapy	7 (16)
Information not available	2 (4)

^*^based on an exact Wilcoxon rank sum test to handle tied observations for comparing the distributions of c-Met expressions between characteristic groups. FNG, Fuhrman nuclear grade.

### c-Met expression is increased in metastases compared to primary tumors and is associated with higher FNG

**Figure 1:**
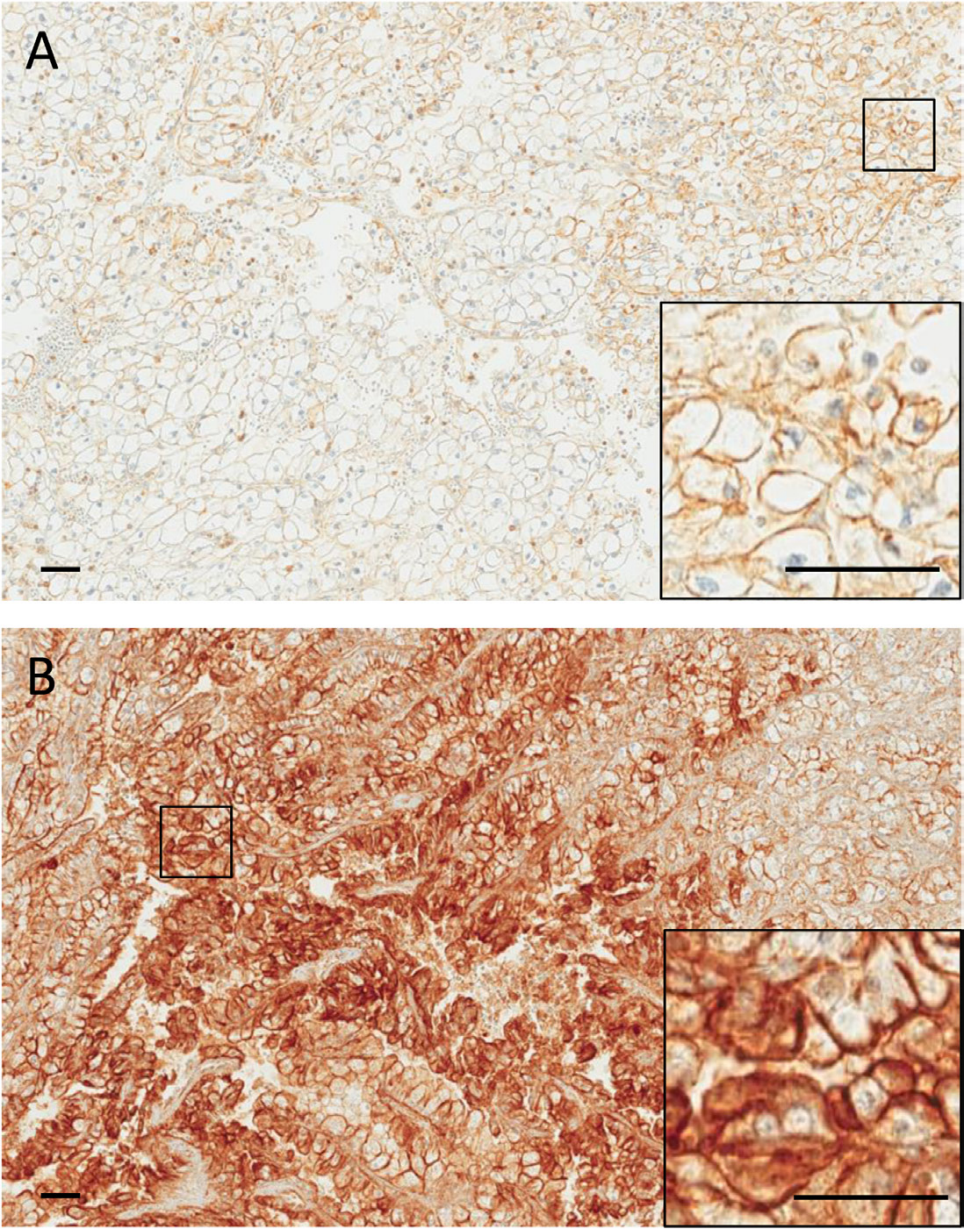
Representative images of a primary ccRCC (**A**) and its corresponding metastasis (**B**) immunostained for c-Met. A, weak (1+) membranous staining is observed in a subset of tumor cells. B, intense (3+) membranous staining is observed in a large fraction of tumor cells. Insets show higher magnifications of the selected areas. Scale bars: 50 µm.

c-Met expression (average c-Met combined score (CS) > 0) was detected in 34 of 45 (75.6%) primary ccRCC samples and 41 of 54 (75.9%) metastases. Overall, c-Met expression levels were higher in metastatic sites compared to paired primary tissues (average c-Met CS (interquartile range, IQR): 55 (30, 83) vs. 28 (10, 55), *p =* 0.0003, respectively) (Figure 1A and 1B). Higher c-Met expression in primary tumors was shown to be associated with higher FNG (4 vs 3; average c-Met CS: 52 vs. 20, *p =* 0.04, respectively) and patients with T-stage T3-T4 appear to have higher c-Met expressions compared to those with Tx-T2 (average c-Met CS: 30 vs. 24, *p =* 0.13, respectively) (Table 2).

**Table 2:** Associations of c-Met expression with FNG, T-stage, and PD-L1 status, according to tumor site

	c-Met expression in primary tumors			c-Met expression in metastatic tumors		
**Characteristic**	***N***	**Median (IQR)**	*p*-value^*^	***N***	**Median (IQR)**	*p*-value^*^
FNG			0.04			0.42
III	32	20 (6,43)		32	53 (14,81)	
IV	13	52 (20,75)		13	60 (35,130)	
T-stage			0.13			0.72
Tx-T2	20	24 (9,38)		20	48 (26,81)	
T3-T4	25	30 (12,70)		25	55 (30,83)	
PD-L1 status			0.34			0.45
(-), ≤ 0%	32	24 (9,46)		36	51 (26,81)	
(+), > 0%	13	30 (12,64)		9	60 (35,130)	

### Association of c-Met expression with PD-L1 expression

**Figure 2:**
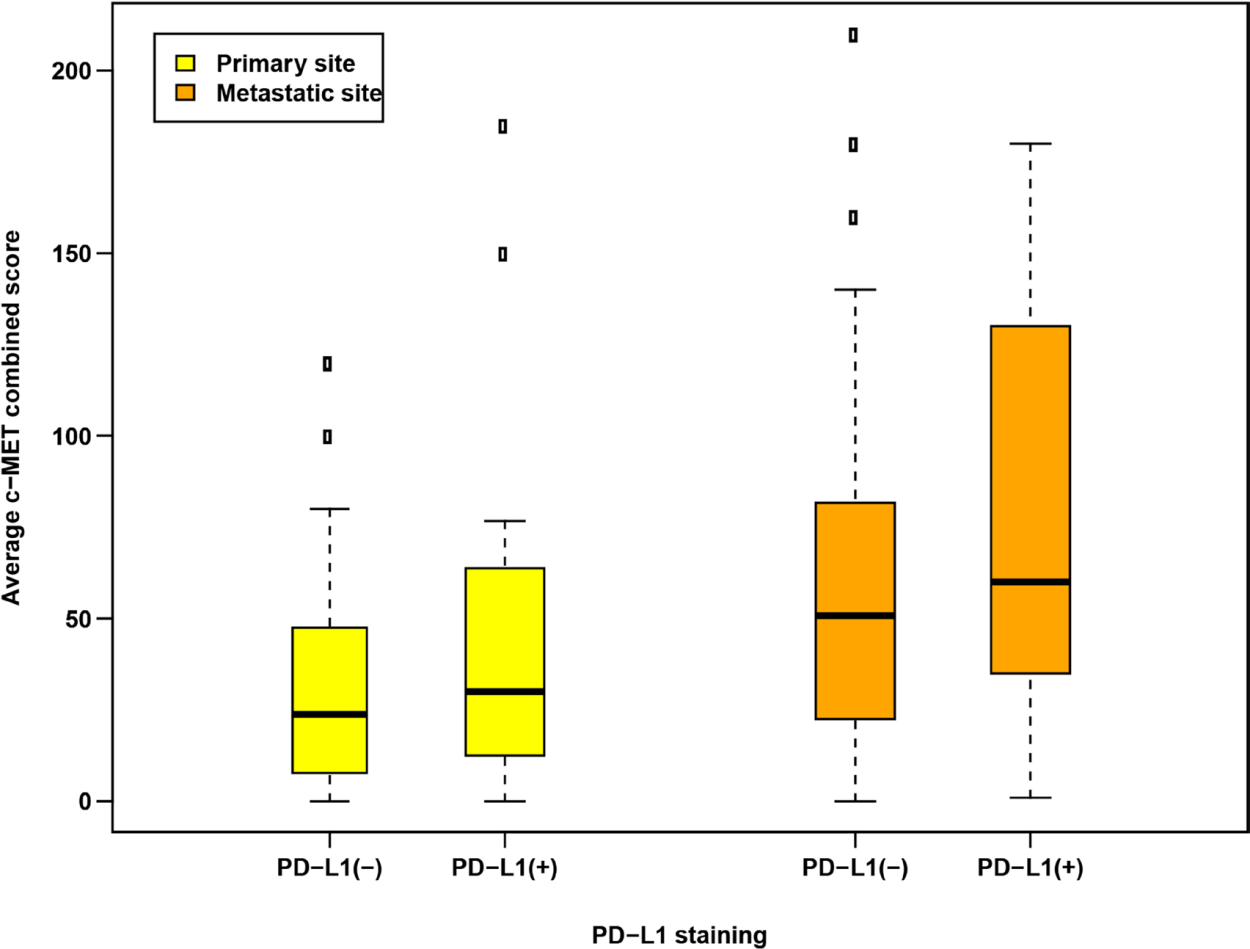
Distributions of c-Met expression according to PD-L1 staining positivity (PD-L1+, > 0% positive cells vs. PD-L1-, = 0% positive cells) based on tumor sample sites

**Figure 3:**
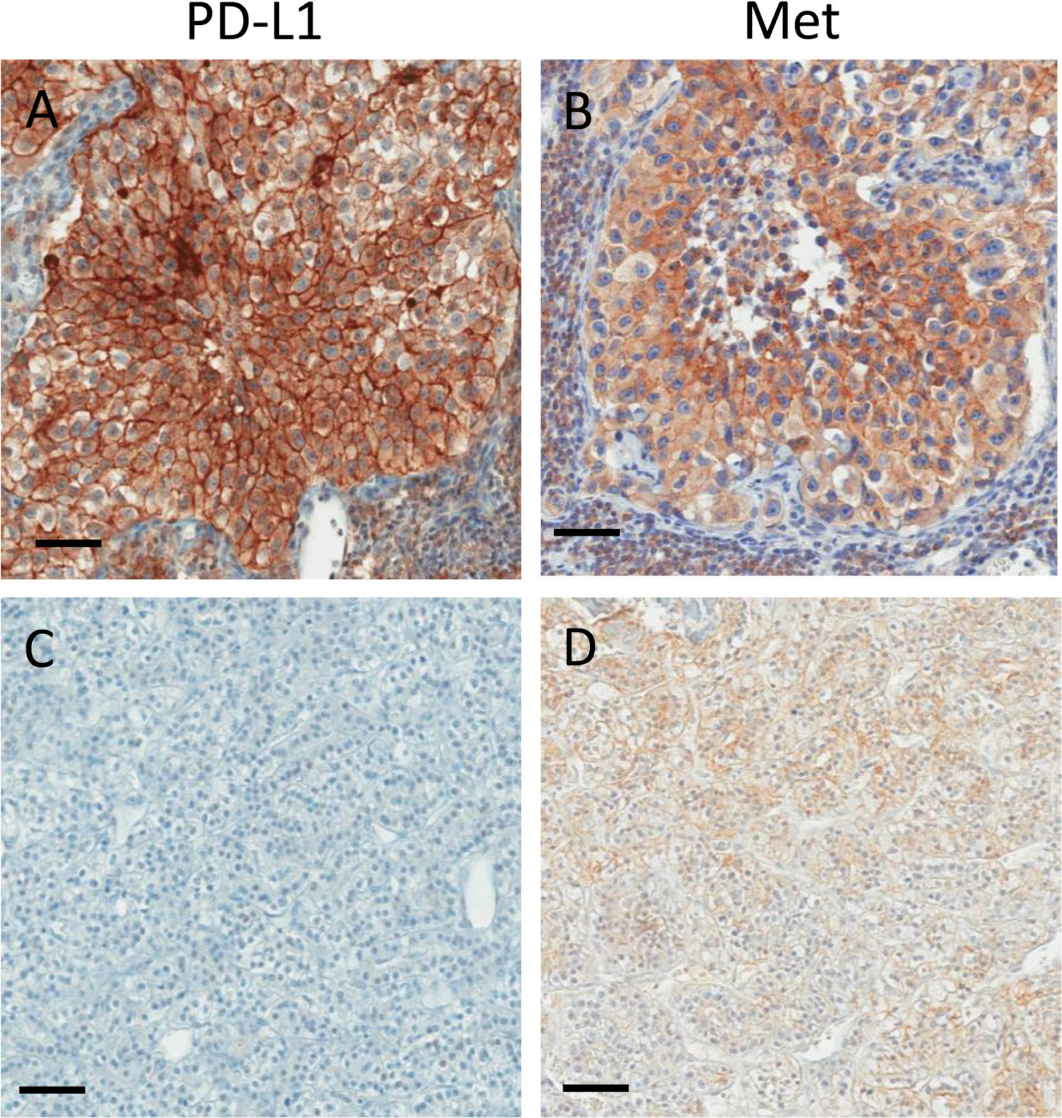
Representative images of two ccRCC metastases immunostained for PD-L1 (**A**, **C**) and c-Met (Met; **B**, **D**). PD-L1 positive metastasis (A) with intense (3+) membranous c-Met staining (B). PD-L1 negative metastasis (C) with weak (1+) membranous c-Met staining (D). Scale bars: 50 µm.

Of the 45 pairs of samples available for c-Met analysis, 13 (29%) displayed PD-L1 positivity in the primary tumor and 9 cases (20%) were PD-L1 positive in paired metastases, including 7 cases (16%) with PD-L1 positivity in both primary tumors and metastases. PD-L1 positive tumors had numerically greater c-Met expression than PD-L1 negative tumors in both primary (average c-Met CS: 30 vs. 24, *p =* 0.34, respectively) and metastatic sites (average c-Met CS: 60 vs. 51, *p =* 0.45, respectively), although these differences were not statistically significant (Table 2, Figure 2, Figure 3A–3D).

### Clinical outcomes by c-Met expression and treatment effect of VEGF-TT on c-Met expression

Forty-three patients had clinical treatment information available. Twenty patients received treatment with VEGF-TT in the first-line setting, 7 received no systemic therapy and 16 were treated with vaccine-based or other therapies. In a descriptive analysis of the 20 patients treated with VEGF-TT, those with high primary c-Met expression had numerically worse time-to-treatment failure (TTF) compared to those with low c-Met expression (estimated median TTF (95% confidence interval, CI): 6.1 (5.3–NA) vs. 9.3 (2.56–NA) months). Similarly, those with high primary c-Met expression had numerically worse overall survival (OS) compared to those with low c-Met expression (median OS (95% CI): 18.1 (12.1–NA) vs. 34.6 (11.4–NA) months).

Among eight patients who received systemic therapy in between surgical resection of their primary and metastatic sites, 6 patients were treated with VEGF-TT. Thirty-five patients had no systemic therapy in between primary and metastases resection. Patients treated with VEGF-TT in between surgical resection of primary tumor and metastases appeared to have greater c-Met expression in their metastatic sites compared to those who had no systemic treatment in between surgeries (average c-Met CS 69.2 vs. 40.0, respectively). In contrast, c-Met expression in primary tumors was similar in patients who received VEGF-TT before resection of metastases and those who received no treatment (average c-Met CS 20.0 vs. 27.5, respectively).

## DISCUSSION

We demonstrate that in our cohort of ccRCC samples, c-Met expression is significantly higher in corresponding metastatic sites compared to paired primary tissues. Given that the treatment landscape for mRCC has expanded to include small-molecule inhibition of VEGFR, MET, and AXL pathways [14–16], our findings are informative in that they suggest that testing for biomarkers of response to c-Met inhibitors should be conducted in metastases. We further note that higher c-Met expression is seen with higher FNG and T-stage, which underscores the key role of the *c-MET* proto-oncogene in tumor development and aggressiveness [1–3].

While c-Met has been shown to be a key player in the development and progression of a variety of solid tumors, it has therapeutic, and potentially diagnostic, relevance in RCC. As a consequence of hypoxia and inactivation of the Von-Hippel-Lindau tumor suppressor gene (VHL), MET and AXL are upregulated [8, 9]. These pathways are also mechanisms for resistance to antiangiogenic therapy [10–13, 23]. This knowledge provides therapeutic rationale for inhibition of these pathways to improve outcomes in RCC, as evidenced by the development of cabozantanib [14–16]. However, identifying biomarkers of response to c-Met inhibitors, in RCC or other cancers, has been elusive. Macher-Goeppinger *et al.* evaluated the prognostic significance of Met molecular status using tissue microarrays (TMAs) of primary tumor and corresponding normal renal tissue samples, and found higher Met expression and copy number associated with dedifferentiation and higher tumor extent. However, 84% of their samples were low-grade (FNG 1 or 2) and only 2 samples overall were FNG 4. Furthermore, they did not evaluate paired metastatic samples compared to the primary RCC tissues [24]. Shuch *et al.* investigated Met expression in matched metastatic and primary ccRCC and found no significant difference between the two sites. Their analysis showed a low level of correlation of Met expressions between the primary and distant tissue (correlation coefficient *R* = 0.5), and their study was limited by a smaller sample size (*n =* 31) [25]. Given that the primary tumor expression did not correlate well with that of the metastases, and knowing that systemic therapy is used to treat metastatic disease, collectively these data and our findings suggest that correlative biomarker analyses for c-Met should utilize metastatic tissue sites.

An interesting finding in our study is that PD-L1 positive tumors were noted to exhibit numerically higher c-Met expression than PD-L1 negative tumors, in both primary and metastatic sites (Table 2, Figures 2, 3 A-D). This is consistent with and builds upon preclinical work by Balan *et al.*, which identified c-Met and PD-L1 to be significantly upregulated and co-localized in renal cancer tissues [19]. In a clinical study of resected primary ccRCC samples, Shin *et al.* show that PD-L1 positivity was significantly associated with increased c-Met expression [26]. Further, Kammerer-Jacquet *et al.* found that in 62 sunitnib-treated mRCC patients with high c-Met expression, 85.5% of these also had PD-L1 overexpression (defined as 2–3 staining intensity) [27]. Collectively, these data suggest a mechanism by which c-Met can promote increased survival of renal cancer cells through the regulation of PD-L1. Molnarfi *et al.* highlight the role of HGF/c-Met pathway as a potential regulator of inflammation and autoimmunity, particularly with immune-related systemic diseases. For example, HGF treatment can increase the levels of PD-L1 expression by dendritic cells and CTLA-4 molecules expressed on T cells, thereby inducing immune tolerance. HGF also markedly alters the antigen-presenting function and differentiation of dendritic cells and interferes with pro-inflammatory NF-κB signaling, thereby inhibiting an inflammatory response [19]. In the context of this biological rationale, our results expand on the interplay between c-Met and PD-L1 as it pertains to human mRCC. While our data require larger, prospective validation, this understanding appears to support the emerging data of c-Met and PD-L1 as rational targets for combination therapeutic trials, such as the ongoing Phase I CaboNivo study (NCT02496208) [28]. With the promise of current and ongoing investigations into c-Met inhibition and PD-1/PD-L1 treatment in RCC, the need for appropriate tissue sampling to facilitate effective biomarker analysis will be important for patient selection and rational clinical trial design.

In a descriptive analysis, we observed that patients in our cohort who were treated with VEGF-TT and had high c-Met expression had numerically worse clinical outcomes, in terms of reduced TTF and OS, compared to those patients treated with VEGF-TT who had low c-Met expression. While our small sample size limited formal statistical testing, our findings are consistent with other data showing that high c-Met expression portends worse clinical prognosis [5, 7, 9, 14, 15]. Furthermore, we observed that patients treated with VEGF-TT in between the resection of their primary RCC and subsequent metastasis had numerically higher c-Met expression in metastatic sites compared to those who did not receive systemic therapy before the resection of the metastases. Plausible explanations may include the treatment effect of agents that predominantly target the VEGFR (such as sunitinib and pazopanib) and the subsequent increase in MET and AXL pathways that have been implicated in developing resistance to VEGFR inhibition [10–13]. Collectively, these findings further highlight the biological rationale for targeting VEGFR, MET, and AXL pathways in the treatment of RCC [14–16].

In this analysis of paired primary and metastatic samples, we used a validated anti-MET monoclonal antibody (MET4 antibody, GFVW, Van Andel Research Institute) and all tumor slides underwent independent pathological review and were scored by an expert genitourinary pathologist (SS). FNG was assessed using established criteria [21]. Nevertheless, our study does have some limitations. Common to most PD-L1 staining reports is the issue of variability in PD-L1 staining methodologies, given the lack of a widely-accepted, common, standard assay. We utilized a PD-L1 positivity definition as > 0% positive cells (i.e. when any tumor cell positivity was detected) given that the correlation between specific PD-L1 levels and inhibition of anti-cancer immunity is not currently well-established and that the optimal cutoff for PD-L1 expression as a biomarker for response to treatment is still unknown. This definition was also used in the context that the pathologist-based evaluation is semi-quantitative and subjective [22]. Finally, our findings would benefit from larger, prospective validation, particularly with respect to the correlation of staining with clinical outcomes and determination of an optimal cutpoint.

In conclusion, we demonstrate that c-Met expression is higher in metastatic sites compared to paired primary tissues in patients with ccRCC. Given the expanding role and ongoing clinical trials of c-Met inhibition in mRCC, our results suggest that the accurate assessment of c-Met expression as a biomarker for these agents should be conducted in metastatic sites. Although the observation of higher c-Met expression in PD-L1+ tumors requires further investigation, it supports the growing rationale for exploring these targets in combination therapeutic trials.

## MATERIALS AND METHODS

### Patients and samples

A cohort of primary ccRCC tumors and the corresponding metastatic sites from 53 patients, who had undergone surgical tumor resections, were identified from two institutions: Brigham and Women’s Hospital and Beth Israel Deaconess Medical Center. Formalin-fixed paraffin-embedded (FFPE) tissue blocks from primary tumor and corresponding regional lymph node or distant metastases were retrieved. For each nephrectomy or metastasectomy specimen, all hematoxylin and eosin-stained slides containing tumor were reviewed by an expert genitourinary pathologist (SS). Fuhrman nuclear grade (FNG) was assessed using established criteria [21]. For each specimen, both areas of highest FNG and areas of predominant nuclear grade were selected for analysis. The study was approved by the local institutional review boards and was conducted in accordance with Good Clinical Practice Guidelines and the Declaration of Helskinki.

### Immunohistochemistry

c-Met expression was evaluated by immunohistochemistry (IHC) using an anti-MET monoclonal antibody (MET4 antibody, GFVW, Van Andel Research Institute) at 1:250 dilution. Out of 53 pairs, 4 cases were excluded for insufficient tumor cells and 4 cases were excluded for failing to meet quality control (QC) for c-Met staining, resulting in 45 pairs that met the QC for IHC reporting. Therefore, the analytic cohort included 45 primary tumors and 54 metastatic sites corresponding to the 45 primary tumors. c-Met expression was measured using a combined score (CS, 0–300) and calculated by: the intensity of c-Met staining (range 0–3) multiplied by the percentage (%) of positive cells (range 0–100).

PD-L1 expression was previously evaluated by IHC and quantified using an H-score, as reported by Callea et al [22]. Specifically, PD-L1 expression was evaluated using a mouse monoclonal anti-PD-L1 antibody (405.9A11) developed by Dr. Gordon Freeman (Boston, MA). The assay was validated using FFPE cell line controls that were known to be either positive or negative for PD-L1 expression by flow cytometry. Tumor sections were stained with the anti-PD-L1 antibody on a Benchmark XT autostainer (Ventana Medical System) with standard antigen retrieval (#950–124, Ventana). An UltraView Universal DAB Detection kit (#760–500, Ventana) was used per the manufacturer’s instruction. Counterstaining was performed as part of the automated staining protocol using hematoxylin (#760–2021, Ventana). Thereafter, slides were washed in soapy water and distilled water, dehydrated in graded alcohol and xylene, mounted and cover slipped. A case was considered positive for PD-L1 when any tumor cell membrane positivity was detected (PD-L1+ defined as PD-L1> 0% positive cells) or else as PD-L1- (= 0% positive cells).

### Statistical analysis

Patient and tumor characteristics at the time of primary surgery were summarized descriptively. When several samples were available within one primary or multiple metastatic sites, an average of c-Met combined score (average c-Met CS) were calculated. Similarly, the PD-L1 expressions according to tumor sites were dichotomized as PD-L1+ (> 0% positive cells) and PD-L1- (= 0% positive cells) in the analysis. The comparisons between paired primary and metastatic samples used Wilcoxon signed-rank test for c-Met expression status (average c-Met CS). The associations of the average c-Met CS with PD-L1 expression (+/–), as well as clinicopathological features of FNG and tumor stage (T-stage) according to the primary and metastatic sites were assessed using the Wilcoxon rank-sum test. In an exploratory analysis of patients treated with first-line VEGF-TT, time to treatment failure (TTF) and overall survival (OS) were estimated using the Kaplan Meier method, according to high c-Met expression levels (defined as c-Met CS ≥ median) versus low (c-Met CS < median). Descriptive statistics were also used to summarize c-Met expression by disease sites (primary vs. metastasis) in patients who had VEGF-TT in between sampling time points compared to those without treatment prior to sampling. All statistical computations were performed using R 13.3.1 (www.r-project.org), and a *p*-value (two-sided) of < 0.05 was considered statistically significant when appropriate.
